# Innovative Approaches to Understanding Resilience

**DOI:** 10.1093/geroni/igaa057.3027

**Published:** 2020-12-16

**Authors:** B Gwen Windham, Michael Griswold

## Abstract

Physical resilience, generally defined as the ability to recover or resist functional decline following a stressor, holds promise for clinicians to identify patients who are likely to benefit from a planned stressor (such as an elective procedure) or deteriorate after an unplanned stressor (such as heart failure). This symposium will present innovative research approaches that enhance understanding of the constructs of resilience from human and animal models. The symposium will include presentations from leading experts addressing issues in the study of resilience including choices of definitions, study design, comparator groups, and considerations of underlying latent factors. Data from population-based cohort studies and animal models will be presented to inform the discussions. This interdisciplinary symposium will address recommendations for advancing resilience knowledge through elucidating biologic, clinical and environmental influences and optimizing definitions, designs, and analyses. Epidemiology of Aging Interest Group Sponsored Symposium.

